# The possible interaction between tryptophan and its metabolites with delirium in older patients with critical illnesses

**DOI:** 10.1007/s41999-024-01114-7

**Published:** 2024-11-27

**Authors:** Korhan Kollu, Huseyin Kurku, Ali Unlu, Busra Ecer, Ibrahim Guney, Muhammet Cemal Kizilarslanoglu

**Affiliations:** 1 Division of Intensive Care Medicine, Department of Internal Medicine, Konya City Hospital, University of Health Sciences Türkiye, Akabe, Adana Çevre Yolu Cd. No: 135/1, 42020 Karatay, Konya, Turkey; 2 Department of Medical Biochemistry, Konya City Hospital, University of Health Sciences Türkiye, Konya, Turkey; 3 Department of Medical Biochemistry, Faculty of Medicine, Selcuk University, Konya, Turkey; 4 Division of Nephrology, Department of Internal Medicine, Konya City Hospital, University of Health Sciences Türkiye, Konya, Turkey; 5 Division of Geriatrics, Department of Internal Medicine, Konya City Hospital, University of Health Sciences Türkiye, Konya, Turkey

**Keywords:** Delirium, Intensive care unit, Kynurenine pathway, Tryptophan, Older patient

## Abstract

**Aim:**

The present study aimed to investigate the relationship between delirium and tryptophan and its metabolites in critically ill older patients.

**Methods:**

This prospective and observational study was conducted on patients who were > 60 years of age and hospitalized for at least 24 h at the internal medicine ICU in the tertiary health care unit (*n* = 120). All consecutively selected patients were evaluated for delirium at the baseline and follow-up period at the bedside by an intensive care specialist. At the end of the 24 h follow up, the patients were divided into two groups (with and without delirium). Clinical properties and tryptophan (TRP) and its metabolites [kynurenine (KYN), kynurenic acid (KYNA), quinolinic acid (QA), 3-hydroxykynurenine (3-HK), 3-hydroxyanthranilic acid (3HAA)] were compared between groups.

**Results:**

The median age of the patients was 79.5 (62–95) years and 53.3% were female. The median age and CCI score were significantly higher among patients with delirium than in those without delirium (*P* = 0.001 and 0.031, respectively). The level of TRP was significantly (borderline) decreased among patients with delirium (*P* = 0.056). The KYN/TRP and QA/TRP ratios were statistically and significantly higher in patients with delirium than those without (*P* < 0.001 and *P* = 0.016, respectively). The best predictive values for detecting delirium were calculated as ≤ 14,100 ng/mL for TRP (AUC: 0.601, *P* = 0.052), > 1.12 for KYN/TRP ratio (AUC: 0.704, *P* < 0.001), and > 0.75 for QA/TRP ratio (AUC: 0.627, *P* = 0.013). The QA/TRP ratio showed independent and borderline significant association with being delirium in multivariable regression analysis (Odds ratio: 2.007, *P* = 0.066).

**Conclusion:**

This study demonstrated that tryptophan and its metabolites obtained within the first 24 h of ICU admission might have predictive value for determining high-risk older patients for delirium.

## Key summary points

**Aim:**

To investigate the relationship between delirium and tryptophan and its metabolites in critically ill older patients.

**Findings:**

The level of TRP was significantly (borderline) decreased among patients with delirium (*p* = 0.056). The KYN/TRP and QA/TRP ratios were statistically and significantly higher in patients with delirium than those without (*p* < 0.001 and *p* = 0.016, respectively). The best predictive values for detecting delirium were calculated as ≤14,100 ng/mL for TRP (AUC: 0.601, *p* = 0.052), >1.12 for KYN/TRP ratio (AUC: 0.704, *p* < 0.001), and > 0.75 for QA/TRP ratio (AUC: 0.627, *p* = 0.013). The QA/TRP ratio showed independent and borderline significant association with being delirium in multivariable regression analysis (Odds ratio: 2.007, *p* = 0.066).

**Message:**

This study demonstrated that tryptophan and its metabolites obtained within the first 24 h of ICU admission might have predictive value for determining high-risk older patients for delirium.

## Introduction

Delirium is defined as a syndrome of acute brain failure caused by the direct pathophysiologic consequence of underlying medical conditions or toxic exposure [1]. According to DSM-5, the diagnostic criteria for delirium contains attention deficit, additional impairments in cognition, fluctuations that develop quickly, and changes in severity throughout the day [2–4]. It may also develop due to the substance deprivation or intoxication [4]. Unlike other psychiatric diseases, delirium cannot be better explained by any pre-existing neurocognitive disorder [3]. Delirium, as a global brain dysfunction, is a complex syndrome that may present with highly variable cognitive impairments within a short time. The heterogeneity of symptoms and clinical course may easily lead to misdiagnosis [5–7]. It is important to note that between 32 and 67% of patients who develop delirium in medical intensive care units (ICUs) have been reported to be unrecognized and overlooked by physicians [8, 9]. Of three clinical types, hyperactive delirium is characterized by restlessness, hyper vigilance, rapid and pressured speech, anger, nightmares, distraction, and euphoria [1, 3]. On the contrary, sleepiness, slowness of movements, decreased awareness, apathy, and decreased arousal are prominent in the hypoactive type. The mixed type presents with alternating symptoms of both [1, 3].

While the pathophysiology of delirium has not yet been thoroughly elucidated, different factors have been implicated in its etiology. Along with precipitating (i.e., trauma, tumors, surgical operations, internalization into ICUs, and metabolic factors) and predisposing factors (i.e., age, cognitive, visual or hearing impairments, frailty, comorbidities), several neurobiologic mechanisms, including neuro-inflammation, destruction of neuronal networks, disequilibrium in neurotransmitter levels, and cerebral vascular dysfunctions have involved in the pathogenesis [3].

As the most common neuropsychiatric disorder among hospitalized patients, the incidence of delirium is vastly variable from 11 to 42% [10] among medical in-patients, which has been reported unchanged over the last four decades [11]. Despite vigorous global efforts to improve routine management in ICUs, delirium incidence has been reduced from 60 to 80% [6, 7, 12] to 31.8% [13] within more than a 15 year time [14]. In addition to increased mortality, delirium is strongly associated with prolonged mechanical ventilation and neurophysiologic dysfunction, thus delayed functional improvement and higher costs ICU patients [5, 6, 15, 16].

Most ICU delirium-prediction models based on clinical factors have shown satisfactory performance, yet with variable predictive ability [17–19]. Besides low clinical applicability, they are not dynamic enough to indicate timely fluctuations in critically ill patients. Although a wide variety of candidate biomarkers have been reported to have predictive value for delirium, the current literature is inconclusive for any biomarker having a predictive value for delirium in critically ill or elderly patients due to the heterogeneity of study designs [20–22]. Moreover, predictive values of biomarkers may differ according to specific clinical conditions such as postoperative delirium (POD) in surgical cohorts or delirium in medical cohorts as acute critically ill patients or critically ill elderly patients [20–27]. As the excess of several neurotransmitters, amino acids, and their by-products [such as acetylcholine, serotonin, dopamine, and gammaaminobutyric acid (GABA)] are usually implicated in the pathophysiology of delirium, identifying a candidate biomarker and its metabolites early at ICU admission may be a reliable risk marker for the impending transition to delirium [28].

A growing body of evidence indicates that imbalances in the level of tryptophan (TRP) and its metabolites are associated with a wide range of human pathologies, including depression, schizophrenia, autoimmunity, neurodegeneration, delirium, cardiovascular diseases, and cancer [29–32]. TRP, one of the large neutral amino acids (LNAAs), can cross the blood–brain barrier and is a precursor to serotonin and melatonin neurotransmitters in the central nervous system (CNS) [33]. Although studies investigating the association between TRP levels and delirium in ICU patients have reported conflicting results, the combined examination of TRP and kynurenine pathway (KP) in early phase of ICU admission may unveil delirium pathophysiology in medical inpatients [20–27, 29]. Two of the six studies found a significant association between high tryptophan levels and postoperative delirium [25, 34] while three studies found significantly low tryptophan levels in patients with delirium [26, 35, 36]. In a study involving orthopedic surgery patients, it was shown that there was no relationship between tryptophan level and delirium [37].

The KYN/TRP ratio is a widely recognized marker of IDO activity and kynurenine pathway engagement. Elevated KYN/TRP ratios indicate that a larger proportion of TRP is being metabolized into KYN, reducing the availability of TRP for serotonin production. As KYN and its downstream metabolites have both neuroprotective and neurotoxic properties depending on the context, increased kynurenine pathway activity has been implicated in various neuropsychiatric and neurodegenerative conditions, including delirium [38].

Likewise, the QA/TRP ratio highlights the extent to which TRP is converted into quinolinic acid, a potent NMDA receptor agonist known to exert excitotoxic effects on neurons. High levels of QA have been associated with neurodegeneration, oxidative stress, and cognitive dysfunction, all of which are common features in delirium [39]. Elevated QA/TRP ratios, therefore, may indicate an increased risk for neuronal damage and cognitive decline in critically ill patients.

Despite these insights, the specific roles of KYN/TRP and QA/TRP ratios in predicting delirium risk in ICU settings have not been fully explored. While TRP levels in isolation may not sufficiently differentiate patients at risk for delirium, these ratios, which capture the metabolic reallocation of TRP within the kynurenine pathway, may offer greater predictive value. Understanding the dynamics of these ratios could help in identifying high-risk patients and potentially guide therapeutic interventions aimed at modulating the kynurenine pathway.

In this study, we aim to investigate the relationship between TRP and its metabolites—particularly the KYN/TRP and QA/TRP ratios—and the development of delirium in critically ill older patients. By assessing these biomarkers within the first 24 h of ICU admission, we seek to determine their potential predictive value for delirium, with the hypothesis that heightened kynurenine pathway activity may be a key factor in the pathogenesis of delirium.

## Materials and methods

### Patients and study design

This prospective and observational study was conducted on patients who were > 60 years of age and hospitalized for at least 24 h at the internal medicine ICU in the tertiary health care unit at the Konya City Hospital. The patients with a diagnosis of severe dementia, active cancer, or end-stage liver disease and with whom communication was impossible (i.e., stupor, coma, intubated, and hearing difficulties) were excluded from the study.

All consented patients were evaluated for delirium at the baseline using the Confusion Assessment Method for ICU (CAM-ICU) [40] and the Diagnostic and Statistical Manual of Mental Disorders (DSM-5) [2] criteria at the bedside by an intensive care specialist. All patients were consecutively selected into the study if not having exclusion criteria and underwent evaluation of delirium criteria described and mentioned above within the 24 h of ICU admission in three different times (8 h intervals). When the delirium was detected, this patient was included in the delirium group after taken blood sample, that is, blood samples were obtained immediately from the delirium cohort. Otherwise, the blood sample was obtained at the end of the first 24 h in those who did not show the clinical picture of delirium.

### General characteristics and evaluated parameters

A comprehensive set of data about general demographic characteristics including age, gender (female, male), ICU outcome (deceased, discharged), with and without delirium according to DSM-5 criteria, hypoactive, hyperactive and mixed type according to DSM-5 delirium subtypes. The patients were enrolled with and without delirium according to CAM-ICU criteria. The comorbidities included coronary artery disease (present/absent), congestive heart failure (present/absent), peripheral vascular disease (present/absent), cerebrovascular disease (present/absent), dementia (present/absent), chronic obstructive pulmonary disease (present/absent), connective tissue disease (present/absent), peptic ulcer (present/absent), liver disease (present/absent), diabetes mellitus (present/absent), hemiplegia (present/absent), severe kidney disease (present/absent), diabetes mellitus with end-organ damage (present/absent), presence of tumors (present/absent), leukemia (present/absent), lymphoma (present/absent), moderate liver disease (present/absent), metastatic solid tumor (present/absent), AIDS (present/absent) were documented. Chronic drug use (present/absent), number of drugs on the first day of ICU admission, education level (illiterate, primary school graduate, secondary school graduate, high school graduate, university graduate), marital status (single, married, widowed), alcohol use (present/absent), and smoking (non-user, active smoker, quitter) were recorded. The delirium risk factors (hearing problems, visual problems, sepsis, acute vascular events, pathologies related to central nervous system, electrolyte imbalance, hypoxia, malnutrition, dehydration, trauma, cancer, history of alcohol use, central venous catheter, and urinary system catheterization) and reason for ICU admission (sepsis, respiratory failure, acute renal failure, gastrointestinal system bleeding, and hypervolemia) were recorded. The APACHE-II [41] and SOFA [42] scores, the Charlson’s Comorbidity Index (CCI) [43], the Glasgow Coma Scale (GCS) were recorded in the patient evaluation form. The malnutrition risk was assessed by Nutritional Risk Screening-2002 (NRS-2002) [44]. The patients who received ≥ 3 points in NRS-2002 were defined as at risk for malnutrition. Serum samples were analyzed by glucose hexokinase method, creatinine and BUN tests by enzymatic kinetic method, albumin, magnesium, and phosphorus tests by colorimetric method, sodium and potassium ion selective electrode method, vitamin B12, vitamin D, folate, ferritin, TSH, free T4, free T3 and procalcitonin were measured by electrochemiluminescence immunoassay (ECLIA) method and CRP test was measured by immunoturbidimetric method using commercial kits with Roche COBAS 8000 (Roche Diagnostics, Mannheim, Germany) automatic analyzer system. Anthropometric measurements were not performed. The levels of TRP and its metabolites were compared between the two study groups.

### Evaluation of tryptophan and its metabolites

#### Sample storage

Blood samples were centrifuged at 4000 g for 10 min. Serum samples were separated into Eppendorf’s and stored at − 80 °C until the measurement day.

#### Mobile phase preparation

Mobile phases A and B were prepared as a gradient with high-performance liquid chromatography (HPLC) grade water containing 0.1% formic acid and acetonitrile containing 0.1% formic acid, respectively. After the prepared mobile phases were mixed well, they were left in an ultrasonic bath at room temperature for 30 min.

#### Precipitant preparation

A 1% formic acid containing acetonitrile solution was prepared to precipitate proteins.

#### Solvent preparation

To dissolve the dried residues remaining after the evaporation step under nitrogen gas, a 25:75 acetonitrile: water solution containing 0.1% formic acid was prepared.

#### Sample preparation for the measurement

Serum samples stored at −80 °C were allowed to thaw at room temperature and then vortexed. 100 μL of internal standard (kynurenine-d4) and 1000 μL of precipitant were added to 300 μL of a serum sample. The solution was vortexed for 30 s and centrifuged at 12,000 rpm for 10 min. After centrifugation, the supernatants were placed in clean glass tubes and dried under nitrogen gas at 40 ℃. Following drying, 250 μL of solvent was added to the tubes and placed in vials.

#### Measurement with LC–MS/MS device

TRP and its metabolites [kynurenine (KYN), kynurenic acid (KYNA), quinolinic acid (QA), 3-hydroxykynurenine (3-HK), 3-hydroxyanthranilic acid (3HAA)] were measured at the Department of Biochemistry at the Medical School in Selcuk University by using a triple quadrupole mass spectrometer (AbSciex API 3200 LC–MS/MS). The precursor to product ion m/z values were 205.2/146.2, 209.1/94.1, 190.2/144.0, 154.0/136.0, 225.1/110.0, 168.0/124.0, and 213.1/140.1 for TRP, KYN, KYNA, 3HAA, 3-HK, QA, and KYN-d4, respectively. The ion-source values for electrospray ionization positive mode were as follows: ion spray voltage, 5000 V; source temperature, 350 °C; curtain, 20 psi; ion source (GS1), 50 psi; and ion source (GS2), 50 psi, respectively. The intra- and inter-assay imprecision values were less than 12% for all analytes [45].

The studies have reported that the frequency of delirium in elderly patients hospitalized in intensive care units can be as high as approximately 87% [7]. Considering this rate, sample size and power were calculated. The analysis was performed through the OpenEpi program. There are 32 intensive care units with 32 beds in our hospital, and it is predicted that the number of patients over the age of 60 who can be admitted to these intensive care units within 3 months may be approximately 180. Therefore, the minimum number of patients to be included in order to reach 5% alpha error (design effect = 1) and 95% confidence interval and power was determined as at least 105 patients.

#### Statistical analysis

Statistical analysis was carried out using the Statistical Package for Social Sciences (SPSS) statistical software package version 21.0. The normal distribution of numerical variables was examined using the Kolmogorov–Smirnov test. Normally distributed numerical variables were expressed as mean ± standard deviation, non-normally distributed numerical variables were expressed as median (minimum–maximum), and categorical variables were expressed as numbers and percentages. Independent group analysis was performed using the Student *t* test or Mann–Whitney U test where appropriate. The Chi-Square or Fischer Exact tests were used to compare categorical data between independent groups. The Spearman’s correlation analysis was used to test the correlations between numerical variable. Multivariable logistic regression analysis was done to show the independently associated parameters for delirium. The receiver operating characteristic (ROC) curve analysis was applied to detect the best cut-off values and sensitivity and specificity levels of the significantly continuous parameters related to the delirium. *p* value < 0.05 was accepted as statistical significance.

## Results

The study included 120 patients admitted to the medical ICU, of whom 58 (48.3%) were diagnosed with delirium (Table 1). The mortality rate was significantly higher among patients with delirium when compared to those without (56.9% vs. 22.6%; *p* < 0.001). According to the DSM-5 criteria, the hyperactive type was observed in slightly more than one-fourth of patients with delirium. No hearing problem, trauma, or acute vascular event was detected in the subjects as a risk factor for delirium. The cause of ICU admission was sepsis in most of the study cohort (44.2% overall and 53.4% in the delirium cohort). The comparison of general clinical features in patients with and without delirium is presented in Table 1.

**Table 1:** Comparison of general clinical features in patients with and without delirium

Parameters	Overall *n* = 120		Without delirium *n* = 62		With delirium *n* = 58		*p* value
	*n*	%	*n*	%	*n*	%	
**Gender**, *female*	64	53.3	29	46.8	35	60.3	0.136
*ICU outcome*							
Discharged	73	60.8	48	77.4	25	43.1	< 0.001
Deceased	47	39.2	14	22.6	33	56.9	
Delirium according to DSM-5	58	48.3	–	–	–	–	–
*Delirium types*							
Hypoactive	17	14.2	–	–	–	–	–
Hyperactive	32	26.7	–	–	–	–	–
Mixed	9	7.5	–	–	–	–	–
Delirium according to CAM-ICU	59	49.2	–	–	–	–	–
*Comorbidities*							
DM	51	42.5	25	40.3	26	44.8	0.618
CAD	45	37.5	24	38.7	21	36.2	0.777
HF	43	35.8	18	29.0	25	43.1	0.108
CVA	34	28.3	18	29.0	16	27.6	0.861
COPD	31	25.8	16	25.8	15	25.9	0.994
Dementia	22	18.3	8	12.9	14	24.1	0.112
*Education level*							
Illiterate	25	20.8	11	17.7	14	24.1	0.632
Primary school	36	30.0	17	27.4	19	32.8	
Secondary school	35	29.2	20	32.3	15	25.9	
High school	24	20.0	14	22.6	10	17.2	
*Marital status*							
Married	73	60.8	43	69.4	30	51.7	0.048
Widow	47	39.2	19	39.6	28	48.3	
*Alcohol intake*							
Never	111	92.5	58	93.5	53	91.4	0.808
Active drinker	3	2.5	1	1.6	2	3.4	
Quitted	6	5	3	4.8	3	5.2	
*Smoking*							
Never	61	50.8	31	50.0	30	51.7	0.644
Active smoker	29	24.2	17	27.4	12	20.7	
Quitted	30	25.0	14	22.6	16	27.6	
*Delirium risk factors*							
Hearing problems	0	0.0	–	–	–	–	–
Visual problems	114	95.0	59	95.2	55	94.8	0.933
Sepsis	51	42.5	21	33.9	30	51.7	0.048
Acute vascular event	0	0.0	–	–	–	–	–
CNS pathologies	20	16.7	9	14.5	11	19.0	0.513
Electrolyte imbalance	32	26.7	16	25.8	16	27.6	0.826
Hypoxemia	46	38.3	22	35.5	24	41.4	0.507
Malnutrition	28	23.3	12	19.4	16	27.6	0.287
Dehydration	39	32.5	17	27.4	22	37.9	0.219
Trauma	0	0.0	–	–	–	–	–
Central venous catheter	105	87.5	54	87.1	51	87.9	0.890
UT catheterization	120	100.0	–	–	–	–	–
*Cause for ICU admittance*							
Sepsis	53	44.2	22	35.5	31	53.4	0.049
Respiratory failure	19	15.8	10	16.1	9	15.5	
Acute pancreatitis	6	5.0	3	4.8	3	5.2	
Acute kidney failure	15	12.5	7	11.3	8	13.8	
GI bleeding	14	11.7	8	12.9	6	10.3	
Hypervolemia	13	10.8	12	19.4	1	1.7	

*ICU* intensive care unit, *DSM*−5 diagnostic and statistical manual of mental disorders 5th edition, *CAM-ICU* confusion assessment method for ICU, *DM* diabetes mellitus, *CAD* coronary artery disease, *HF* heart failure, *CVA* cerebrovascular accident, *COPD* chronic obstructive pulmonary disease, *CNS* central nervous system, *UT* urinary tract, *GI* gastrointestinal

The median age of the delirium cohort was 82 (range: 64–95) years, and 60.3% were female (Table 2). CCI score was significantly higher among patients with delirium (*p* = 0.031). The medians of clinical prognostic scoring systems and vitamin B12 levels were significantly higher in the delirium cohort (*p* < 0.001) (Table 2).

**Table 2:** Comparison of numerical parameters between the study groups

Parameters	Overall *n* = 120 median (min.–max.)	Patients without delirium *n* = 62 median (min.–max.)	Patients with delirium *n* = 58 median (min.–max.)	*p* value
*General clinical characteristics*				
Age, years	79.5 (62–96)	74 (62–96)	82 (64–95)	0.001
CCI	6 (2–14)	5 (3–12)	7 (2–12)	0.031
Number of chronic medications	1 (0–1)	1 (0–1)	1 (0–1)	0.344
Number of medications on the first day of ICU	7 (5–12)	8 (5–12)	7 (5–11)	0.207
*Clinical scoring*				
APACHE-II	24 (11–42)	22 (11–37)	27.5 (14–42)	< 0.001
GCS	13 (8–15)	14 (8–15)	13 (8–15)	< 0.001
SOFA	6 (2–15)	5 (2–12)	7 (2–15)	0.001
*Laboratory tests*				
Glucose, mg/dL	150 (74–699)	153 (86–358)	139 (74–699)	0.178
BUN, mg/dL	35 (8–111)	34 (8–111)	46 (10–109)	0.123
Creatinine, mg/dL	1.15 (0.32–10.04)	1.03 (0.32–10.04)	1.25 (0.37–4.28)	0.500
Sodium, mmol/L	139 (121–167)	139 (126–167)	139 (121–160)	0.632
Potassium, mmol/L	4.1 (2.3–6.6)	4.1 (3–6.6)	4.2 (2.3–5.1)	0.444
Magnesium, mg/dL	1.87 (1.22–3.86)	1.87 (1.22–3.86)	1.87 (1.23–3)	0.867
Phosphorus, mg/dL	3.5 (1.1–12.1)	3.1 (1.1–12.1)	3.8 (1.6–7.3)	0.204
Calcium, mg/dL	7.95 ± 0.85	7.98 ± 0.82	7.93 ± 0.89	0.750
Albumin, g/dL	28 (18–45)	29 (18–45)	27 (20–41)	0.139
Hemoglobin, g/dL	10.2 ± 2.12	10.19 ± 2.34	10.2 ± 1.89	0.987
WBC, 10^3^/µL	11.6 (0.09–66.58)	11.47 (0.09–39.14)	11.75 (1.33–66.58)	0.574
Neutrophil, 10^3^/µL	9.74 (0.02–78.9)	9.79 (0.02–29.81)	9.53 (0.11–78.9)	0.891
Lymphocyte, 10^3^/µL	0.88 (0.06–10.6)	0.81 (0.06–2.98)	0.93 (0.26–10.6)	0.392
Monocyte, 10^3^/µL	0.64 (0.01–4.25)	0.62 (0.01–1.55)	0.65 (0.01–4.25)	0.520
Platelet, 10^3^/µL	202 (7–536)	185.5 (7–536)	211.5 (54–484)	0.277
Vitamin B12, ng/L	444.5 (209–1109)	566.5 (230–980)	325.5 (209–1109)	< 0.001
Folate, µg/L	5.52 (1.54–20)	5.48 (1.54–20)	5.57 (1.72–18.2)	0.858
Ferritin, µg/L	310 (9–2163)	254 (9–2000)	354.5 (21–2163)	0.580
Vitamin D, ng/L	4.62 (1–41.8)	3.53 (1–32.9)	5.1 (1.17–41.8)	0.168
TSH, mU/L	1.14 (0.01–19.9)	1.14 (0.02–6.09)	1.12 (0.01–19.9)	0.940
free T4, ng/L	12.4 (0.39–32.2)	12.4 (0.39–24.7)	12.7 (1.07–32.2)	0.279
free T3, ng/L	1.6 (0.39–8.49)	1.62 (0.4–3.96)	1.57 (0.39–8.49)	0.610
CRP, mg/L	88.95 (0.9–508.11)	72.02 (0.9–508.11)	104.25 (1.99–391)	0.086
Procalcitonin, µg/L	0.41 (0.03–100)	0.42 (0.04–100)	0.39 (0.03–100)	0.578

*CCI* Charlson comorbidity index, *ICU* intensive care unit, *APACHE*-II acute physiology and chronic health evaluation-II, *GCS* glasgow coma scale, *WBC* white blood cell, *SOFA* sequential organ failure assessment, *BUN* blood urea nitrogen, *TSH* thyroid-stimulating hormone, *CRP* C-reactive protein

**Fig. 1:**
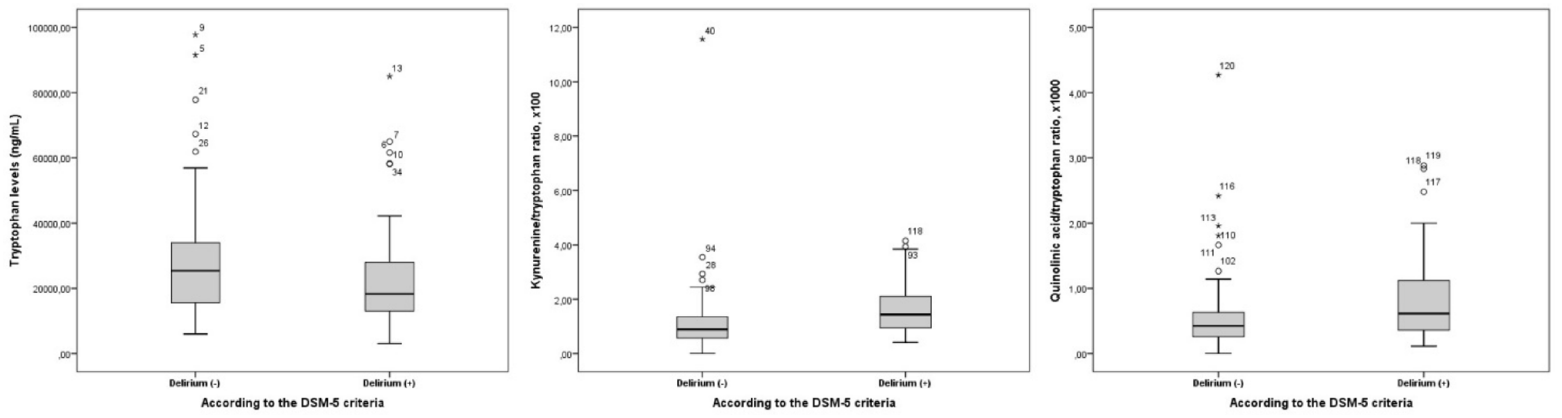
Comparison of the levels of tryptophan and its metabolites between patients with and without delirium according to DSM-5 criteria

The level of TRP was significantly (borderline) decreased among patients with delirium (*p* = 0.056). The KYN/TRP and QA/TRP ratios were statistically and significantly higher in patients with delirium than those without (*p* < 0.001 and *p* = 0.016, respectively) (Table 3 and Fig. 1). When compared with the numerical variables, TRP and its metabolites showed significantly negative correlations with thyroid stimulating hormone (TSH), free T4, GCS, monocyte count, platelet count, and vitamin D levels (Table 4). Significantly positive correlations were determined between TRP and its metabolites and folate, free T3, CCI score, sodium levels, and the number of medications.

**Table 3:** Comparison of tryptophan and its metabolites between the study groups

Tryptophan and its metabolites median (min.–max.)	Overall cohort *n* = 120	Patients without delirium *n* = 62	Patients with delirium *n* = 58	*p* value
TRP, ng/mL	21,150 (3110–97800)	25,400 (6000–97800)	18,250 (3110–85000)	0.056
KYN, ng/mL	233.5 (25.8–1990)	215 (25.8–1990)	252.5 (48.5–1590)	0.217
KYNA, ng/mL	7.54 (2.32–66.7)	7.43 (2.32–66.7)	7.58 (3.17–19.2)	0.996
3HAA, ng/mL	14.6 (1.37–194)	15.95 (1.37–143)	13.85 (2.4–194)	0.844
3-HK, ng/mL	3.2 (1.03–51.6)	3.21 (1.06–42.3)	3.16 (1.03–51.6)	0.777
QA, ng/mL	10.9 (5.48–88.5)	11.35 (5.49–88.5)	10.65 (5.48–29.4)	0.860
KYN/TRP ratio, × 100	1.12 (0–11.57)	0.89 (0–11.57)	1.44 (0.41–4.15)	< 0.001
QA/TRP ratio, × 1000	0.5 (0–4.27)	0.43 (0–4.27)	0.61 (0.11–2.88)	0.016

*TRP* tryptophan, *KYN* kynurenine, *KYNA* kynurenic acid, 3HAA 3-OH anthranilic acid, 3-HK 3-OH Kynurenine, *QA* quinolinic acid

**Table 4:** The list of numerical parameters showing significant correlations with tryptophan and its metabolites is presented in this table

Parameters			Rho	*p* value
free T4	vs	TRP	−0.191	0.047
Folate	vs	TRP	0.182	0.047
GCS	vs	KYN	−0.187	0.041
Monocyte	vs	KYNA	−0.212	0.020
TSH	vs	KYN/TRP ratio	−0.222	0.020
free T3	vs	KYN/TRP ratio	0.244	0.011
CCI score	vs	QA/TRP ratio	0.200	0.029
Sodium	vs	QA/TRP ratio	0.182	0.047
Platelet	vs	QA	−0.249	0.006
The number of medications	vs	3-HK	0.261	0.004
Vitamin D	vs	3HAA	−0.293	0.019

*TRP* tryptophan, *GCS* glasgow coma scale, *KYN* kynurenine, *KYNA* kynurenic acid, *QA* quinolinic acid, 3-HK 3-OH Kynurenine, 3HAA 3-OH anthranilic acid

**Fig. 2:**
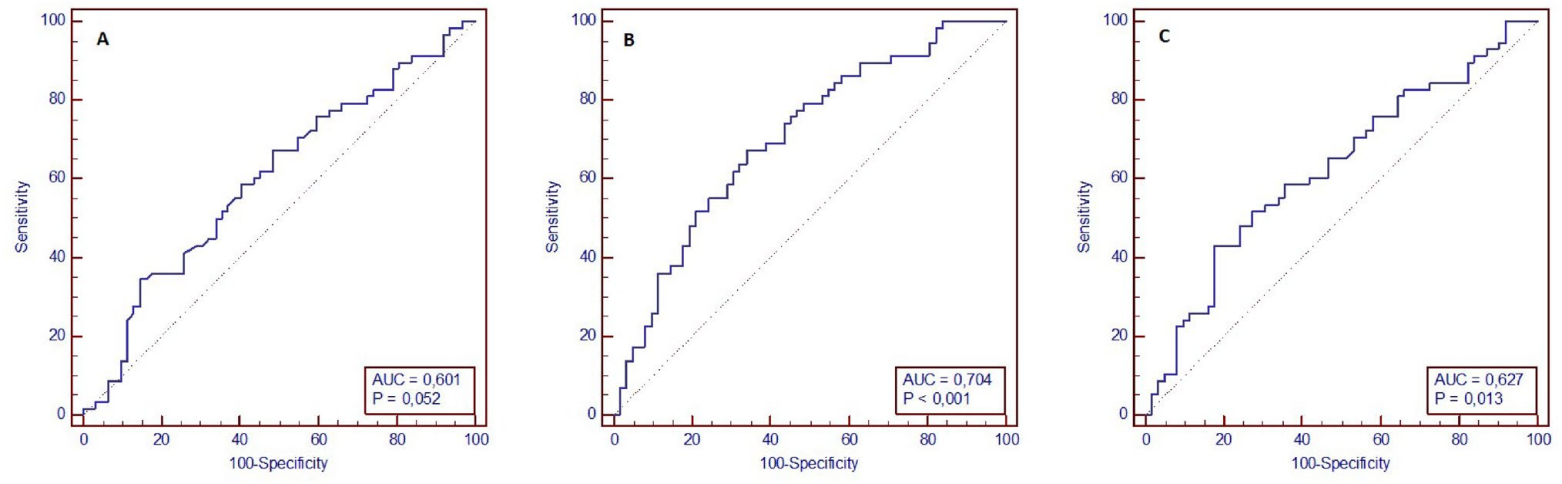
ROC analysis for delirium prediction for **a** tryptophan, **b** kynurenine/tryptophan ratio, and **c** quinolinic acid/tryptophan ratio

The best predictive values for delirium were calculated as ≤ 14,100 ng/mL for TRP (AUC: 0.601, *p* = 0.052; 34.5% sensitivity, 85.5% specificity), > 1.12 for KYN/TRP ratio (AUC: 0.704, *p* < 0.001; 67.2% sensitivity, 66.1% specificity), and > 0.75 for QA/TRP ratio (AUC: 0.627, *p* = 0.013; 43.1% sensitivity, 82.3% specificity) (Fig. 2).

The ROC analysis identified the best predictive cutoff value of tryptophan levels for delirium according to DSM-5 criteria in our patient cohort as ≤ 14,100 ng/mL. Based on this value, all participants in the study were categorized into two groups with low and normal tryptophan levels. A 2 × 2 contingency table analysis of delirium incidence by tryptophan levels showed that the group with low tryptophan levels (*n* = 20/29, 69.0%) had a significantly higher frequency of delirium compared to the group with normal levels (*n* = 38/91, 41.8%) (*p* = 0.011).

In multivariable logistic regression analysis, it was found that age (OR: 1.120 *p* = 0.001), APACHE-II score (OR: 1.163 *p* < 0.001), vitamin B12 levels (OR: 0.991 *p* < 0.001), and QA/TRP ratio (OR: 2.007 *p* = 0.066) were independently associated parameters with delirium (Table 5).

**Table 5:** Multivariable binary logistic regression analysis showing the independently related factors for delirium diagnosed by the DSM-5 criteria

Parameters	Odds ratio	95% confidence interval		*p* value
		Lower limit	Upper limit	
Age, years	1.120	1.047	1.198	0.001
APACHE-II score	1.163	1.070	1.263	< 0.001
Vitamin B12 levels, ng/L	0.991	0.988	0.995	< 0.001
QA/TRP ratio, × 1000	2.007	0.955	4.218	0.066

Multivariable logistic regression analysis was done to show the independently associated parameters for delirium. All parameters showing significantly association (*p* < 0.05) with delirium in univariate analyses were included in multivariable analysis (age, having sepsis, CCI, marital status, APACHE-II score, vitamin B12 levels, KYN/TRP and QA/TRP ratios). Since the APACHE-II score was significantly correlated with the GCS (rho = −0.661) and SOFA (rho = 0.781) scores, only APACHE-II score was added to the regression models. Backward method was used. The last step (step-5) was presented in the table. The Hosmer–Lemeshow Test’s p-value was 0.087, Omnibus test’s *p* value was < 0.001 and Nagelkerke *R*^2^ was 0.573 for this step

The levels of TRP and its metabolites were compared between patients survived and dead in the ICU follow-up period. There were no significant differences between mentioned parameters in terms of ICU mortality (for TRP, *p* = 0.390; for KYN, *p* = 0.554; for KYNA, *p* = 0.547; for 3HK, *p* = 0.807; for 3 HAA, *p* = 0.160; for QA, *p* = 0.919; for KYN/TRP ratio, *p* = 0.126; for QA/TRP ratio, *p* = 0.276).

## Discussion

Delirium is a common, serious, and often fatal condition among older patients in medical ICU settings. To the best of our knowledge, the present study is the first clinical study showing the relationship between serum levels of TRP and its metabolites, and transition to delirium in older ICU patients with severe acute medical conditions. It was shown in this study that lower TRP levels and increased up regulation of KP downstream metabolites (KYN/TRP and QA/TRP ratios) in the blood samples obtained during ICU stay showed significant correlations with the development of delirium. And also, our study results indicate inverse association between KYN levels and GCS, as well as direct association between the QA/TRP ratio and CCI, which denotes that KP indicates progression to a severe possible outcome at the molecular level in clinically critically ill patients. Furthermore, our study is consistent with previous studies that increased age, higher APACHE II, and SOFA scores are risk factors for delirium [46–48]. Moreover, significant correlations between GCS and KYN, as well as CCI and QA/TRP ratio in the delirium cohort, might indicate as early signals for the worst clinical end-point.

As disturbances in the neurotransmitter pathway contribute to the transition to delirium, TRP, the essential amino acid and precursor of serotonin, has been investigated in the pathogenesis of delirium [24, 25, 30, 49, 50]. However, a recent meta-analysis has reported inconsistent results in using amino acids as biomarkers [20]. Evidence from basic research indicates that indoleamine 2,3-dioxygenase (IDO)-dependent TRP metabolism is strongly activated under inflammation [38]. KYN/TRP ratio in the blood is the best marker for measuring deregulated TRP breakdown, including infections, autoimmune disorders, cancer, cardiovascular, and psychiatric diseases [38]. Upregulated KP leads to the production of potential neurotoxic metabolites, one of which is QA [51]. Recent studies have reported confirmatory data about the expanded role of KP downstream metabolites, mainly in neurologic diseases [39].

Our study showed significant differences in KYN/TRP and QA/TRP ratios between the delirium and non-delirium groups, with higher ratios indicating a greater risk for developing delirium. The ROC analysis supported the predictive value of these ratios, especially the KYN/TRP ratio with an AUC of 0.704, suggesting moderate predictive accuracy.

Previous studies investigating a predictive biomarker in patient serum yielded inconsistent outcomes as they were focused on the impact of amino acid levels on the transition to delirium, excluding the KP [25] or investigators on the impact of KP and its metabolites on a combined outcome [52] or in the surgical cohort [53]. Up-to-day, the predictive values of many biomarker candidates have been investigated predominantly for post-operative delirium at the surgical ICU with increased activation of inflammatory cascade due to surgery [23, 24, 37]. In the current study, the overall cohort constituted of non-surgical patients admitted to the ICU to overcome the confounding effects of surgical interventions on the metabolic state. We believed that constituting a cohort of ICU patients with underlying mainly systemic inflammation (i.e., sepsis, acute renal failure, respiratory failure) would provide an investigation of a better mechanistic link with the neuroinflammatory process in delirium.

The relevance of these findings is underscored by the growing recognition that inflammation-driven dysregulation of TRP metabolism is a key mechanism in cognitive disorders, including delirium. By focusing on the ratios that reflect kynurenine pathway activation, rather than on TRP levels alone, this study aligns with the broader literature suggesting that metabolic shifts in TRP utilization are more indicative of delirium risk in critically ill patients [39, 53].

While previous studies have explored the role of TRP and its metabolites in delirium, many have focused on TRP levels without considering the metabolic shifts toward kynurenine production [25, 26]. Our study’s novel contribution lies in its focus on the KYN/TRP and QA/TRP ratios as predictive markers, adding nuance to the understanding of how TRP metabolism impacts delirium. These findings are consistent with studies linking increased KP activity with cognitive dysfunction in other neuroinflammatory conditions [3, 38], further supporting the idea that targeting the kynurenine pathway could be beneficial in preventing or mitigating delirium.

Furthermore, although not related to the main hypothesis of our manuscript, as shown in Table 4, we tried to show the interactions of TRP and its metabolites to the other laboratory variables measured in the study. As seen in the table, their correlation coefficients were weak.

The current study has some limitations. First, we did not perform sequential measurements of KP metabolites during the ICU stay, which would have characterized the association between changes in the KYN/TRP ratio over time, as delirium might have a clinically fluctuating nature. Another limitation is the inability to measure patients’ dietary tryptophan intake due to the variability of dietary needs.

## Conclusion

Tryptophan and KP metabolites obtained within the first 24 h of ICU admission have predictive value for determining high-risk patients for delirium. When combined with clinical risk scoring scales, TRP and its metabolites may be helpful in quick risk stratification for critically ill patients. Further studies including large sample size are needed to elucidate more accurate findings in this area.

## Data Availability

The datasets generated and/or analyzed during the current study are available from the corresponding author on reasonable request.
